# Temperature constrains diet-induced plasticity in the life-history of an aquatic vertebrate

**DOI:** 10.1038/s41598-026-55894-y

**Published:** 2026-06-08

**Authors:** Sara Bento, Emma Deeks, Joana Martelo, Maria F. Magalhães, Pedro Segurado, Sandra Nascimento, Pedro P. Sousa, João Caramelo, Joana Vilas Boas, Anssi Laurila, Richard Svanbäck, Vladimíra D. Carreira, Pavel Kratina, Rui Rebelo, Bruno M. Carreira

**Affiliations:** 1 CE3C – Centre for Ecology, Evolution and Environmental Changes and CHANGE – Global Change and Sustainability Institute, Faculty of Sciences, University of Lisbon, Ed. C2 Campo Grande, 1749-016 Lisboa, Portugal; 2 Centre for Biodiversity and Sustainability, School of Biological and Behavioural Sciences, Queen Mary University of London, Mile End Rd, London, E1 4NS UK; 3 Department of Zoology, Cambridge Research Institute, The University of Cambridge, Cambridge, UK; 4 TERRA Associated Laboratory, Forest Research Centre, School of Agriculture, University of Lisbon, Tapada da Ajuda, 1349-017 Lisboa, Portugal; 5 School of Tourism and Marine Technology, Polytechnic Institute of Leiria, Campus 4, Rua do Conhecimento nº4, 2520-614 Peniche, Portugal; 6 Department of Ecology and Genetics, Section of Animal Ecology, Evolutionary Biology Centre, Uppsala University, Norbyvägen 18D, 75236 Uppsala, UK; 7 MARE – Marine and Environmental Sciences Centre, Department of Environmental Sciences and Engineering, NOVA School of Science and Technology, NOVA University Lisbon (FCT NOVA), 2829-516 Caparica, Portugal

**Keywords:** Ectotherms, Functional traits, Metabolism, Nutritional quality, Dietary shifts, Climate warming, Temperature-size rule, Amphibians, Ecology, Ecology, Physiology, Zoology

## Abstract

Temperature is a major force governing ectotherm life-history. To buffer temperature-driven nutritional imbalances, consumers may engage in selective feeding, thereby altering trait expression and life-history configuration as a consequence of trait-specific energetic and nutritional optima. Yet, the joint effects of temperature and diet on life-history architecture remain poorly understood in vertebrate consumers. We reared *Bufo spinosus* tadpoles under three temperatures (12 °C, 16 °C, 20 °C) and three diet types (animal-based, plant-based, or choice between both), assessing how selective feeding influences trait performance and life-history plasticity. Increasing temperature altered life-history, accelerated larval development and growth, reducing body mass and condition (SMI), with marginal effects on body length. Dietary effects were trait-specific and temperature-dependent, with the animal diet performing better at 12 °C both in growth rate and body mass but losing this advantage at higher temperatures. Tadpoles offered the choice diet fed selectively, leading to lower assimilation of animal-based at higher temperatures, and generally outperformed tadpoles fed fixed diets. However, selective feeding only partially offset the impacts of higher temperatures. Our results show that increasing temperature canalized life-history trajectories, constraining diet-induced plasticity, and highlight the need to integrate thermal and nutritional drivers in predicting ectotherm responses to climate change.

**Supplementary Information:**

The online version contains supplementary material available at 10.1038/s41598-026-55894-y.

## Introduction

Temperature exerts a major selective pressure on ectotherms, and climate warming is expected to profoundly impact their ecology, physiology, and behaviour^1–3^. The expression of life-history traits across a temperature gradient can be described by thermal performance curves, where performance rises to a plateau at the optimum temperature, with steeper decreases towards high than low temperatures^4,5^. However, because traits scale differently with temperature, warming can lead to imbalanced responses. For instance, the “temperature-size rule” (TSR) was proposed after the repeated observation of a stronger influence of temperature on developmental rate compared to growth rate, promoting earlier maturation and reducing adult body size^6^. Additionally, warming accelerates ectotherm metabolic rates, raising maintenance costs and nutrient demands^7,8^ while feeding rates and assimilation efficiency may fail to increase proportionally under stressful or supra-optimal temperatures^9–11^. This often results in mismatches between nutrient demand and intake, which can lead to interactive effects on trait performance driven by diet-dependent responses to temperature^12,13^. To sustain the nutritional demands imposed by temperature, ectotherms can adjust energy allocation among the traits composing their life-history, leading to distinct configurations of trait covariance^6,7^. However, the extent to which diet modulates temperature effects on the combination of life-history traits, i.e. trait architecture, remains poorly understood.

The influence of temperature on the nutritional ecology of ectotherms adds a layer of complexity to the known effects of temperature on their life-history^14^. Following differences in thermal performance curves among taxa^15,16^ this may produce taxon-specific diet-dependent responses. For instance, moderate temperatures and balanced diet C: N ratios often boost growth rate^17^ and body mass, whereas low temperatures coupled with carbon-rich diets tend to suppress growth^9,17–19^. Higher temperatures coupled with nitrogen-rich diets can accelerate development^20–23^, often at the cost of decreased survival or life span^20,24,25^, and intensify the negative effects of imbalanced diets, reducing body size^18,20,21^. Overall, warming exacerbates nutritional stress in invertebrates by narrowing the range of nutrient ratios that support optimal trait performance^21,22^. Empirical evidence in vertebrate consumers is still limited, but emerging patterns suggest similar diet-dependent thermal responses. For example, carbon-rich diets coupled with higher temperatures increased body mass at maturity in Atlantic salmon, *Salmo salar*^26^, and larval growth in anuran amphibians^27^, whereas nitrogen-rich diets slowed anuran larval growth^27^.

To offset temperature-driven changes in nutritional status, ectotherm consumers can use selective feeding. Recent empirical evidence shows that lower temperatures increase the preference for nitrogen-rich diets, whereas moderate warming promotes a switch to more carbon-rich diets in insects^25^, crustaceans^28,29^, gastropods^30,31^, fish^32,33^ and amphibians^27^. This trophic plasticity enables consumers to buffer thermal stress but may impact the specific configuration of their life-history, because the performance of individual traits is maximized at distinct thermal and nutritional combinations^34^. Selective feeding can protect the structural integrity of the life-history architecture shaped by evolutionary history and may lead to developmental canalization around traits critical for individual fitness, such as body size^35,36^.

Investigating the interplay between temperature- and diet-driven effects is crucial to understand their role in structuring the architecture of ectotherm life-history traits. Here, we experimentally examined the independent and combined effects of experimental temperatures and diet type on the life-history traits of a model vertebrate ectotherm. We reared tadpoles of the Spiny toad, *Bufo spinosus*, at three experimental temperatures (12 °C, 16 °C, 20 °C) on three diets (animal-based, plant-based; or both, henceforth designated as choice), comparing the plasticity of life-history traits (larval growth and developmental rate, and toadlet body mass, length, and condition (SMI) at metamorphosis). We also examined changes in whole body C:N ratio and whether selective feeding, traced with stable isotopes, could buffer the negative effects of warmer temperatures. We hypothesize that temperature strongly shapes the life-history trait architecture of our model species, and that the magnitude of effects is trait-specific and diet-dependent. Following the TSR, increasing temperature should accelerate developmental and growth rates, reducing body mass, length, and condition at metamorphosis. According to ectotherm nutrition ecology, trait performance on the animal-based, nitrogen-rich diet should be highest under cooler conditions, while on the plant-based, carbon-rich diet it should be highest at higher temperatures. Tadpoles allowed choice should feed selectively according to temperature, increasing the plant-based assimilation at warmer temperatures, and outperform individuals on animal- and plant-based diets.

## Materials and methods

### Animal welfare

Collection of *Bufo spinosus* tadpoles was authorized under ICNF licence no. 821/2022/CAPT. All procedures were conducted in accordance with Portuguese and European legislation governing the care and use of animals in research (Decreto-Lei n. º 113/2013; Directive 2010/63/EU), after approval by the Animal Welfare Body of the University of Lisboa. Tadpoles and toadlets used for stable isotope analyses were euthanized by rapid cooling followed by freezing, a method validated for small-bodied ectothermic vertebrates^37–40^ and used to avoid chemical contamination of isotopic signatures. Sex was not determined because external phenotypic sex cannot be reliably assessed at metamorphosis.

### Model species

The spiny toad, *Bufo spinosus* (Daudin, 1803), is widely distributed across the western Mediterranean basin^41^, a known climate change hotspot^42,43^. Occurring in a diverse range of habitats^44^, its bottom-dwelling omnivore tadpoles feed on algae, detritus and invertebrates, ranking among the most carnivorous anuran larvae in the Iberian Peninsula^45,46^. Like other bufonid larvae, *B. spinosus* tadpoles are gregarious, and social context can influence their activity and feeding behaviour, with consequences for growth^47,48^. Larvae of this species withstand a wide thermal range (CTmin = 3 °C; CTmax = 38 °C) and have been reared successfully at temperatures ranging from 9 °C to 27°C^49–53^.

### Animal collection and experimental design

Newly hatched tadpoles of *B. spinosus* (Gosner stages 21–24^54^) were collected on 13 May 2022 from five points along the littoral zone of a permanent pond in Serra de Sintra (204 m a.s.l.), central Portugal (38°47′27.91′′N, 9°25′10.23′′W). Tadpoles were transported to the animal facility at the Faculty of Sciences of the University of Lisbon and acclimated at 12 °C in eight 6-L aquaria (ca. 40 individuals per aquarium), under a 12 L:12D photoperiod, until 26 May 2022. During this period, tadpoles were fed ad libitum every other day with dehydrated powdered lettuce following water renewal. This temperature was selected to reflect pond water temperatures during the breeding season and to slow and standardise development. Because hatchlings were still transitioning to independent feeding^54^, food was provided only as a precaution during this holding period.

The experiment followed a full-factorial design with nine treatments (three temperatures × three diets). To maintain a biologically relevant social context while keeping density constant across treatments, each treatment consisted of 15 cups, each holding two tadpoles (*N* = 30). Although sub-optimal (but see *Data analysis*), this paired design preserved and prioritised social exposure, thereby improving animal welfare conditions. Test temperatures (12 °C, 16 °C, and 20 °C) were selected to cover a broad, ecologically relevant gradient within the species’ thermal range reported for the larvae of this species, including cold winter-spring and warmer developmental conditions, while avoiding deleterious temperatures. Diet treatments included an animal-based diet (A; nitrogen-rich, freeze-dried Chironomidae larvae), a plant-based diet (P; nitrogen-poor, commercial spinach), and a choice diet (C; simultaneous offer of equal amounts of A and P food items), allowing selective feeding. Each food item was ground into a fine powder, reconstituted in water-insoluble alginate gel (0.02 g mL^− 1^), and solidified into pellets with a solution of calcium chloride (0.25 M), thereby standardizing food shape, texture, and access to digestible contents. The pellets assembled for each food item differed in C:N ratio and isotopic signature (Table S1).

### Experimental procedures and response variables

Before entering the experiment, tadpoles (Gosner stage 25^54^) were individually photographed to determine body length (BL; distance between the snout tip and insertion of the tail in the body) and total length (TL; distance between the snout tip and the tail tip) using ImageJ software. Afterwards, tadpoles were weighed (± 0.1 mg) to determine initial body mass, transferred in pairs to 0.4-L transparent plastic cups and randomly assigned to treatments. Tadpoles did not differ significantly across treatments (Table S2).

Each cup contained a fibreglass net attached to a river stone to simulate macrophyte structure, which increased habitat complexity and provided refuge. Cups were fitted with a lateral mesh-covered opening to permit water flow, and a netted bottom to allow faecal deposition and prevent coprophagy. Cups were transferred into one of six 6-L transparent boxes, each with an even representation of diet treatments, and placed in thermostatic incubators (Lovibond TC 445 S, Germany; ±0.5 °C), operated with the same shelving, lighting and ventilation configuration. During the experiment, tadpoles were fed *ad libitum* every other day, after removal of food leftovers and water renewal, with cup positions randomized within each box, and box positions randomized within thermostatic incubator.

Tadpole survival was monitored daily, and death events were recorded throughout the experiment until metamorphosis, characterized by forelimb emergence (Gosner stage 42^54^). Developmental rate was assessed as time to metamorphosis, expressed as the number of days (hereafter designated as larval period). At this stage, metamorphosing tadpoles were transferred to 50-mL plastic cups containing a thin water layer and a small stone, where they remained until tail resorption (Gosner stage 46^54^). Toadlets were photographed against a millimetric scale to determine body length (distance between the snout tip and the urostyle; mm) and weighed (± 0.1 mg). Larval growth rate was expressed as mean daily mass gain (mg day^− 1^; Gosner stages 25–46^54^). Body condition was estimated using the scaled mass index (SMI)^55^, with an average BL of 13.95 mm (b_SMA_ = 3.63). ﻿Body mass, body length, and body condition were analysed as complementary descriptors of metamorphic phenotype, respectively representing total biomass, structural size, and standardised mass.﻿

### Stable isotope analysis

The proportion of plant- and animal-based food items assimilated by tadpoles on the choice diet was estimated using stable isotope analysis. For this, we used a subsample of toadlets, including the first individuals to complete metamorphosis on each cup (*N* = 131). Individuals were euthanized by rapid cooling followed by freezing, avoiding chemical contamination of isotopic signatures^37^. This procedure has been previously validated^38,39^ and conforms with ethical principles for small (body mass < 4 g) ectothermic vertebrates^40^. Individuals were degutted, oven-dried at 60 °C for 24 h, homogenised to a fine powder, and samples with 1.0 ± 0.2 mg were weighed into tin capsules (Elemental Microanalysis^®^ 6 × 4 mm) for analysis. The determination of carbon (δ^13^C) and nitrogen (δ^15^N) stable isotope ratios and C:N elemental ratios was conducted at Queen Mary University of London, UK, using an Isotope Ratio Mass Spectrometer (Sercon Integra2 EA–IRMS). Protein IRMS Standard (Elemental Microanalysis^®^ OAS/Isotope 5 g) was inserted after every 10 samples. Isotopic ratios were expressed in standard delta notation in parts per thousand (‰).

### Data analysis

The statistical analysis focused on testing the fixed and interactive effects of temperature and diet on survival, life-history traits and tissue C:N ratio, as well as comparing the estimated proportion of plant- and animal-based items assimilated by individuals on the choice diet. Box and cup identity were included as separate random intercept terms in all analyses to account for the non-independence arising from the experimental design.

Survival was analysed using Cox proportional hazards mixed-effects models (*coxme* package^56^. Temperature and diet were included as fixed effects with initial body mass (*wi*) as a covariate, and inference was based on likelihood-ratio tests between nested models. Temperature × diet interactions were not tested due to complete separation issues arising from zero mortality events in one treatment combination (20 °C on Animal diet), preventing the reliable estimation of interaction effects. Model assumptions were evaluated using residual diagnostics from a corresponding fixed-effects Cox model (*survival* package^57^. Proportional hazards were violated for temperature due to differences in larval period among temperatures; therefore, temperature effects on survival are interpreted descriptively, with likelihood-ratio tests used as supportive inference.

For continuous traits, we fitted generalised linear mixed models (GLMMs) with error structures matching the distribution of each response variable, using the *glmmTMB* package^58^. Growth rate, body mass and C: N ratio were fitted using Gamma GLMMs (log-link function); larval period and body condition were fitted using Gaussian GLMMs on log-transformed responses; and body length was fitted using a Gaussian GLMM on the original scale. Box identity was removed as a random intercept from the body condition model because its negligible variance caused convergence issues due to overparameterization. Initial body mass (*wi*) was included as a covariate to account for individual-level variation in size, while the proportion of the larval period that each tadpole spent with its pair (*S*) accounted for variation in social exposure following the death or metamorphosis of its pair (Table S3). The *S* covariate was excluded from the larval-period analysis to avoid circularity issues, since it is mathematically dependent on larval period. Model adequacy was evaluated by inspection of the residuals using the *DHARMa* package^59^. Strong deviations from the expected distribution were observed in the residuals of the body condition (SMI) model and addressed by explicitly modelling residual dispersion as a function of temperature and diet using the *dispformula* argument in *glmmTMB*. Inference on the fixed and interactive effects of temperature and diet was obtained using Type II Wald χ² tests (Anova, *car* package^60^. The relative magnitude of these effects within each trait was compared by quantifying effect sizes using hierarchical semi-partial marginal R^2^. When a random intercept prevented stable estimation of R^2^ (e.g., growth rate, body mass and body size: box identity σ^2^ ≈ 0), values were computed from an otherwise identical model excluding that component; this simplification was only used for effect-size calculation and did not affect inference.

Because tadpoles within each pair were not individually identifiable throughout the larval period, initial body mass records were assigned by matching the larger initial tadpole to the first metamorphosing individual. This assignment was based on every other day monitoring during the experiment, over which the size difference in each tadpole pair remained consistently distinguishable. The robustness of this assumption was tested by repeating all analyses using 10,000 random within-pair permutations of initial body mass records (Table S4). Treatment-level inference was qualitatively unchanged, with strongly supported effects consistently recovered and only borderline effects showing the expected sensitivity around the significance threshold. Therefore, we retained the observation-based assignment for the final analyses. Post-hoc pairwise comparisons were performed using the *emmeans* package^61^ with Bonferroni correction. Model predictions fitted on the log scale were back-transformed. To visually compare temperature- and diet-driven plasticity across traits, the model-estimated means of each treatment were normalised to the highest mean within each trait (0–100 scale).

The proportion of plant- and animal-based diet assimilated by tadpoles on the choice diet was estimated using Bayesian stable isotope mixing models, which offer reliable and time-integrated assimilation estimates^62,63^. Model assumptions were met, with the food items differing considerably in C:N ratio, and by more than 2‰ in the isotopic signature of either carbon or nitrogen (Table S1). Furthermore, the half-life values of ^13^C and ^15^N turnover rates obtained for consumers within the same range of body mass (100 mg–1 g) at 10–20 °C suggest that tadpole isotopic signatures were at equilibrium at the end of the larval period^64^. The MixSIAR package was used in the analysis for its ability to handle complex and realistic scenarios, prediction accuracy^65^ and increasing application in diet tracing^63,66,67^. The isotopic signatures of tadpoles reared on single-diet treatments (animal- or plant-based) were used as reference to estimate temperature- and diet-specific trophic enrichment factors (TEFs)^68^. We ran a MixSIAR model per temperature (12 °C, 16 °C, and 20 °C), using the isotopic signatures of tadpoles fed on the choice diet, the corresponding TEFs, and the isotopic signature of the food items as inputs. Models were fitted with both process and residual error terms, and without concentration dependence. MCMC estimation used the “extreme” MixSIAR settings, providing long chains for robust posterior sampling. Convergence was assessed using Geweke and Gelman-Rubin diagnostics^69,70^; all models showed* R*^2^ values below recommended thresholds and only minor deviations in Geweke z-scores, with narrow credible intervals indicating strong model fit. Posterior distributions of the two food items were compared pairwise among temperatures. Differences were summarized using 95% Bayesian credible intervals (BCI) and the posterior probability of a negative difference^71^. All statistical analyses were conducted using R version 4.5.1^72^.

## Results

### Larval survival, growth, and developmental rate

Temperature did not significantly influence mortality risk over time in the Cox mixed-effects model (*P* = 0.11; Table 1; Fig. S1), even though overall survival to metamorphosis increased from 12 °C (75.6%) to 20 °C (97.8%). Diet significantly affected mortality risk (*P* = 0.032; Table 1; Fig. S1), which on the choice diet was lower compared to the plant diet (*P* = 0.039), and marginally lower compared to the animal diet (*P* = 0.072). Overall survival to metamorphosis was highest on the choice diet (90%) compared with both single-diets (81–82%).

**Fig. 1:**
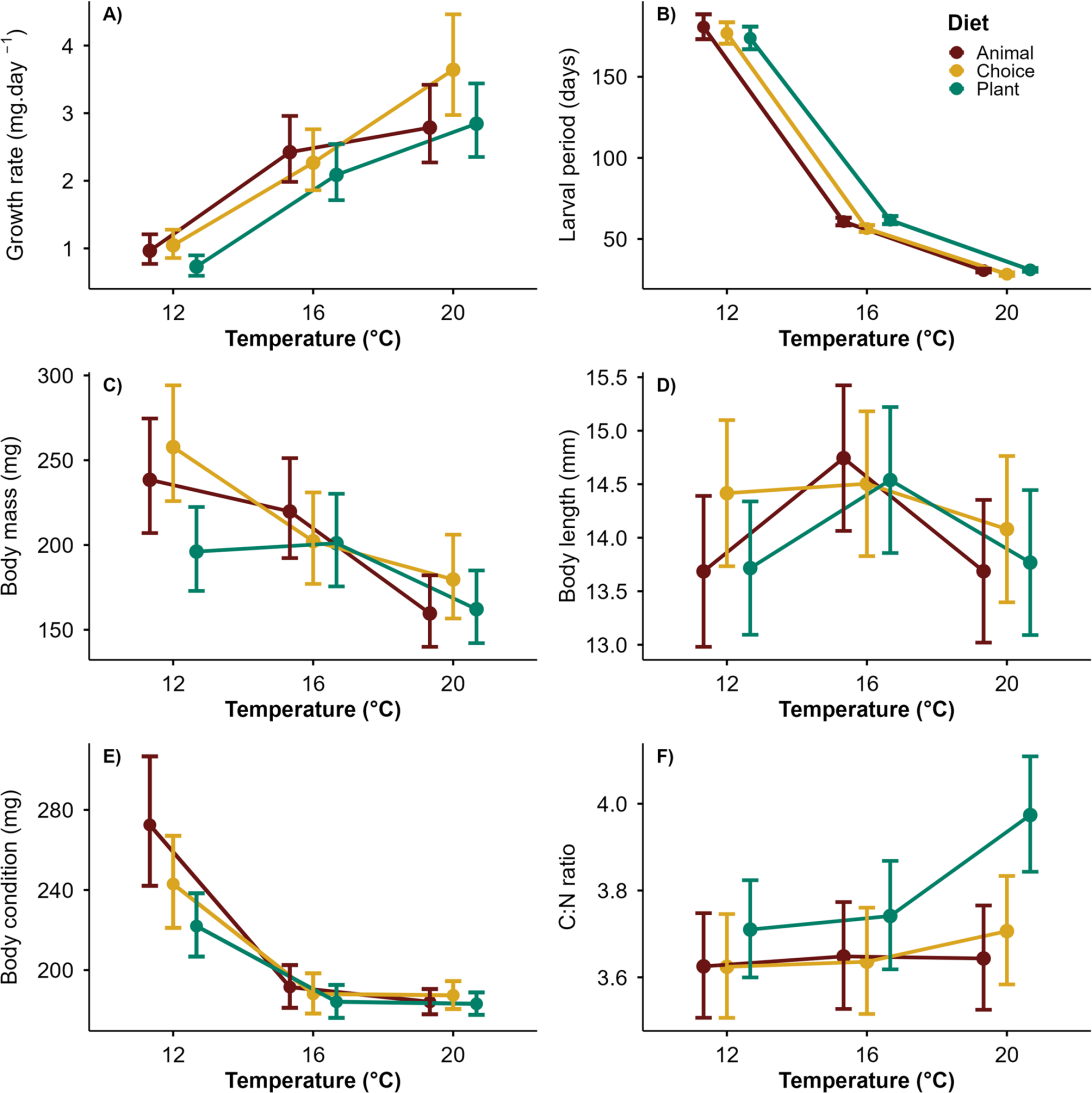
Model-estimated means (± 95% confidence intervals) for six life-history traits of *Bufo spinosus* across temperature and diet treatments. Panels show: (**A**) Growth rate (mg day^−1^), (**B**) Larval period (days), (**C**) Body mass (mg), (**D**) Body length (mm), (**E**) Body condition (mg; standardised body length = 13.95 mm), and (**F**) Whole-body tissue C:N ratio. Colours denote diet treatments: animal (dark red), choice (yellow), and plant (teal). Values correspond to the best-supported models identified by AICc. Temperature treatments were 12 °C, 16 °C, and 20 °C.

Growth rate increased with temperature (*P* < 0.001; R^2^ = 0.674; Table 1; Fig. 1A), being 3.4× higher at 20 °C (3.10 ± 0.10 mg day^− 1^) compared to 12 °C (0.91 ± 0.03 mg day^− 1^). Diet effects on growth rate depended on temperature, as indicated by the significant temperature × diet interaction (*P* = 0.005; Table 1; Fig. S2). Growth rate at 12 °C was lower on the plant diet compared to the animal and choice diets (*P* < 0.01 in both cases), and growth rate at 20 °C was higher on the choice diet compared to the animal and plant diets (*P* < 0.01 in both cases; Fig. 1A). At 16 °C, growth rate did not differ significantly among diets. Growth rate increased with temperature in each diet, except on the animal diet, where growth rate was similar at both 16 °C and 20 °C.

Larval period decreased significantly with temperature (*P* < 0.001; R^2^ = 0.982; Table 1), showing a six-fold reduction from 12 °C (177.1 ± 2.7 days) to 20 °C (29.9 ± 0.4 days) (*P* < 0.001 for all comparisons; Fig. 1B). Diet effects on larval period depended on temperature, as indicated by the significant temperature × diet interaction (*P* = 0.004; Table 1; Fig. S2). Diet did not affect larval period at 12 °C, whereas at 16 °C and 20 °C tadpoles on the choice diet had shorter larval periods compared to those on either the animal or plant diets (both *P* < 0.01 at each temperature; Fig. 1B).

### Toadlet body mass, length and condition

Body mass decreased with temperature (*P* < 0.001; R^2^ = 0.385; Table 1), declining 27.2% from 12 °C (233.9 ± 5.3 mg) to 20 °C (170.3 ± 3.7 mg; Fig. 1C). Diet effects on body mass depended on temperature, as indicated by the significant temperature × diet interaction (*P* < 0.001; Table 1; Fig. S2). Diet effects on body mass were evident only at 12 °C, for which body mass was higher on the animal and choice diets compared to the plant diet (*P* < 0.01 in both cases), whereas no diet differences were detected at 16–20 °C (Fig. 1C). Additionally, body mass on the choice diet was higher at 12 °C compared to both 16 °C and 20 °C, whereas on the animal and plant diets body mass was higher at 12 °C and 16 °C compared to 20 °C (Fig. 1C), with declines of 33.0% on the animal diet and 17.3% on the plant diet.

Body length was affected by temperature (*P* < 0.001; R^2^ = 0.182; Table 1), peaking at 16 °C (14.42 ± 0.1 mm) and being higher compared to both 12 °C (13.76 ± 0.1 mm; *P* < 0.001) and 20 °C (13.66 ± 0.1 mm; *P* < 0.001). Diet had only a small effect on body length (*P* = 0.047; R^2^ = 0.065; Table 1; Fig. S2). The marginally significant temperature × diet interaction (*P* = 0.057; Table 1) suggested temperature-specific diet differences. At 12 °C, toadlets from the choice diet had greater body length compared to those from the plant and animal diets (both *P* < 0.05; Fig. 1D). At 16 °C and 20 °C, body length did not differ significantly among diets. Additionally, body length on the animal and plant diets peaked at 16 °C being higher compared to both 12 °C and 20 °C (all *P* < 0.01; Fig. 1D), whereas this temperature pattern was not observed on the choice diet (both *P* > 0.256).

Body condition decreased with temperature (*P* < 0.001; R2 = 0.432; Table 1), declining 24.5% from 12 °C (245.0 ± 7.0 mg) to 20 °C (184.9 ± 1.9 mg; Fig. 1E). Diet (*P* = 0.190) and the temperature × diet interaction (*P* = 0.104) were not significant (Table 1; Fig. S2), even though a difference in body condition was observed between animal- and plant-fed toadlets at 12 °C, with higher values in the animal diet group (*P* < 0.05; Fig. 1E). No diet differences in body condition were detected at 16–20 °C.

**Table 1:** Summary statistics of the Cox proportional hazards regression, LMMs and GLMMs used to analyse the survival rate, the life-history traits (growth rate, larval period, body mass, body length, body condition), and whole-body C:N ratio of *Bufo spinosus*.

	Temperature				Diet				Temperature × Diet			
	df	*X* ^2^	*P*	*R* ^2^	df	*X* ^2^	*P*	*R* ^2^	df	*X* ^2^	*P*	*R* ^2^
Survival rate	2	4.36	0.113	–	2	6.91	**0.032**	–	–	–	–	–
Growth rate	2	695.7	**< 0.001**	0.674	2	23.9	**< 0.001**	0.036	4	14.8	**0.005**	0.013
Larval period	2	7266.3	**< 0.001**	0.982	2	27.9	**< 0.001**	0.002	4	15.6	**0.004**	0.001
Body mass	2	109.7	**< 0.001**	0.385	2	17.7	**< 0.001**	0.110	4	20.3	**< 0.001**	0.060
Body length	2	32.3	**< 0.001**	0.182	2	6.10	**0.047**	0.065	4	9.13	0.057	0.038
Body condition	2	80.5	**< 0.001**	0.432	2	3.33	0.190	0.052	4	7.69	0.104	0.030
C: N ratio	2	31.2	**< 0.001**	–	2	98.6	**< 0.001**	–	4	36.2	**< 0.001**	–

*P* < 0.05 are shown in boldface type.

df, degrees of freedom; χ^2^, Wald chi-square statistic;* P*,* P*-value; R^2^, hierarchical marginal R^2^.

### Tissue C:N ratio and diet assimilation

Whole-body tissue C:N ratio increased with temperature (*P* < 0.001; Table 1), being higher at 20 °C (3.83 ± 0.02) compared to 12 °C (3.71 ± 0.02) and 16 °C (3.73 ± 0.02) (*P* < 0.001 in both cases; Fig. 1F). Diet had a significant effect (*P* < 0.001; Table 1), with C:N ratio being higher on the plant diet compared to the animal (4.6%) and the choice diets (4.3%) (both *P* < 0.001; Fig. 1F; Table 1). A significant temperature × diet interaction (*P* < 0.001; Table 1) showed that tissue C:N ratio did not differ across temperature on the animal diet, but increased at 20 °C compared to both 12 °C and 16 °C on the plant diet (both *P* < 0.001; Fig. 1F), and increased at 20 °C compared to 12 °C on the choice diet (*P* < 0.05; Fig. 1F).

**Fig. 2:**
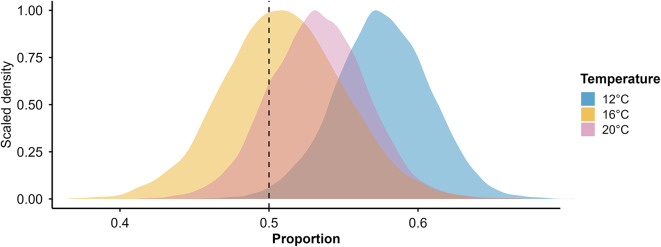
MixSIAR posterior probability density distributions of the proportion of animal-based food assimilated by tadpoles in the choice-diet treatment 12 °C, 16 °C, and 20 °C. The dashed vertical line at 0.5 indicates equal contribution of animal- and plant-based food items.

Tadpoles on the choice diet assimilated relatively balanced proportions of animal (0.54) and plant food items (0.46). Bayesian posterior probability density distributions indicated that assimilation of animal-based food was lower at 16–20 °C compared to 12 °C, although this response was non-linear. At 12 °C, the assimilated proportions of plant and animal food items clearly differed, with the latter having a higher contribution of 0.576 (BCI = 0.513–0.638; Fig. 2). At 16 °C, the contribution of animal food decreased to 0.509 (BCI = 0.431–0.591), with an 89.8% posterior probability of decrease compared to 12 °C (Fig. 2). At 20 °C, the contribution of animal food was 0.533 (BCI = 0.469–0.598), with a posterior probability of decrease of 83.0% compared to 12 °C, and a 67.7% posterior probability of increase compared to 16 °C. Additionally, the δ^15^N values were higher at 12 °C compared to both 16 °C (*z* = 5.97, *P* < 0.001) and 20 °C (*z* = 5.57, *P* < 0.001), and the δ^13^C values at 20 °C were lower compared to both 12 °C (*z* = -10.80, *P* < 0.001) and 16 °C (*z* = -10.75, *P* < 0.001).

## Discussion

Our experimental study demonstrates, for the first time, trait-specific plasticity in the dietary modulation of temperature effects on key life-history traits in vertebrate ectotherms. Temperature governed the life-history of our model species, with increasing temperature accelerating larval development and growth rate, while reducing toadlet body mass and condition at metamorphosis, consistent with the TSR. Diet played a less important and more variable role, exerting modest effects on growth rate and body mass, and having only a limited influence on larval development. Body length was notably fixed, exhibiting minimal variation across temperature and diet treatments. Trait performance under cold conditions was higher on the animal-based diet compared to the plant-based diet, with increased growth rate, body mass, and body condition, but declined sharply at higher experimental temperatures. This likely explains the increased assimilation of plant-based food resources at higher temperatures compared to 12 °C, despite the nonlinear response, suggesting that temperature-dependent dietary shifts toward herbivory may hold adaptive value. These results show that higher temperatures canalise life-history, reducing diet-induced plasticity and imposing constraints to the structuring of life-history trait architecture. Selective and compensatory feeding reduced variation in body length at metamorphosis, underscoring the importance of nutritional context in shaping ectotherm life-history architecture.

### The effects of temperature and diet on life-history traits

Consistent with our hypothesis, we found three distinct forms of trait plasticity. Specifically, a high plasticity to both temperature and diet (growth rate and body mass), limited plasticity to diet (larval period and condition) and reduced plasticity to either temperature or diet (body size). These findings provide novel evidence of trait-specific changes in the breadth of the diet-induced plasticity previously documented in the thermal performance of life-history traits in vertebrate ectotherms^27^. By examining the relative contributions of temperature- and diet-driven trait plasticity, this study advances the mechanistic understanding of how diet modulates the thermal performance of life-history traits. Similarly, previous studies on invertebrates showed that the nutritional quality of food resources alters the magnitude and direction of thermal reaction norms for fitness components in both terrestrial^20,22,25,73,74^ and aquatic ectotherms^17,23,75^.

As expected, temperature overwhelmingly governed larval period, reducing development time by approximately six-fold over a range of 8 °C. Likewise, temperature strongly influenced growth rate, but less markedly. Because of these asymmetric effects, toadlet body mass dropped sharply under higher experimental temperatures. This illustrates the classic TSR^6,76–78^, which is known to be especially strong in aquatic ectotherms owing to the warming-induced reduction in water oxygen concentration^79^. Despite the overall smaller magnitude of the effects, diet also altered life-history trait performance, especially under cold conditions (Fig. S2). For instance, diets with animal food (A and C) increased growth rate and body mass under cold conditions, and the more balanced diet (C) increased growth rate under warm conditions. As shown in previous studies with invertebrates^20,22^, the remarkable variation in the extent to which diet modulated temperature effects suggests that diet-induced plasticity is trait-specific. Hence, thermal performance curves may constrain the breadth of dietary responses, such as selective feeding, and limit their efficiency in accommodating metabolic imbalances imposed by temperature. Furthermore, our findings support that high temperatures reduce the efficiency with which diet can buffer temperature effects on ectotherms, channelling life-histories into a narrower trajectory with diminishing benefits from diet plasticity. This pattern is consistent with recent work showing climate warming to reduce both the expression and effectiveness of plasticity in life-history traits^80^.

Both temperature and diet induced only limited variation in toadlet body length, which consistently peaked at 16 °C across diet types. According to classical developmental theory^81,82^, tadpoles can delay metamorphosis until reaching a minimum threshold for body length, after which surplus energy is channelled into body mass and condition. Here, tadpoles of *B. spinosus* appeared to capitalise on the extended larval period under cold conditions to accumulate reserves and increase body condition, a pattern also reported for *Discoglossus galganoi* under cold conditions^83^. The fixed body length at metamorphosis has also been reported in other amphibians^84,85^ and may constitute a central pillar in the life-history architecture of many anurans. This form of developmental canalization around an optimal body length is common in other ectotherms^86^ and suggests that fixing this morphometric trait may limit the effects of diet-induced plasticity in other life-history traits.

### Temperature-diet interactions and life-history trait performance on the single diet types

The interactive effects of temperature and diet on almost all measured traits, except survival and body condition, support our prediction that diet modulates temperature effects on ectotherm life-history, consistent with the evidence gathered over the last decade^14^. Although the strongest interactions involved the choice diet, interactive effects were also observed in the two single-diet treatments, particularly under cold conditions. At 12 °C, the animal-based diet boosted performance by increasing tadpole growth rate, as well as toadlet body mass and body condition compared to the plant-based diet. These responses align with evidence that ectotherms more efficiently exploit the high nutritional value of nitrogen-rich diets under cold conditions. The low metabolic and gut passage rates enhance nutrient digestion and assimilation^87^, offsetting the costs associated with the digestion of complex macromolecules^88^, and boosting energy conversion into storage tissues (e.g., fat and lean mass). However, the benefits of the nitrogen-rich diet for trait performance declined with increasing temperature, suggesting that higher temperatures constrain nutrient processing and thereby reduce its functional advantages to ectotherm performance.

Contrary to our predictions, warmer temperatures did not enhance trait performance on the plant diet. The similar effects of the animal- and plant-based diets on tadpole life-history traits at higher temperatures indicate a marked narrowing of the performance gap observed under cold conditions, consistent with evidence that higher temperatures diminish or eliminate the advantages of nitrogen-rich diets^27^. Overall, this suggests a strong influence of diet quality on energy storage and allocation at low temperatures, which is increasingly overridden by metabolic and physiological constraints as temperature increases. The lack of evidence for improved trait performance on the plant-based diet at warmer temperatures may partly reflect limited thermal stress across the experimental temperature range (12–20 °C). This interpretation is supported by the positive effect of higher temperature on survival, which has also been reported in other bufonids^89,90^. Nevertheless, the strong developmental canalisation observed here suggests that the lower survival under cold conditions may simply result from the extended larval periods at 12 °C, which increase the chance of mortality events. Additionally, the difference between the C:N ratios of the animal- and plant-based diets was relatively small (~ 50%) compared with the up to eightfold differences reported in previous studies^27,29,31^, and a more imbalanced C:N ratio in the plant-based diet could have amplified its effects. Likewise, alternative animal-based foods might have produced quantitatively different responses owing to resource-specific composition and digestibility. Nevertheless, our interpretation is grounded on the qualitative changes in the efficiency of the animal-base diet and supported by the Geometric Stoichiometry nutritional framework, which accounts for the energetic and metabolic costs of protein catabolism^88^. Furthermore, it is consistent with evidence that temperature alters ectotherm nutrient demands, resource use and the relative value of animal- and plant-based foods in various ectotherms^12,17,27,29,30,83^. Diet-induced plastic responses may have occurred in the gastrointestinal tract, including changes in intestinal length and structure, as diet composition can alter gut morphology in amphibian larvae^91–93^. Such plasticity may have enhanced the assimilation efficiency of the experimental diets, potentially reducing the strength of diet effects on trait performance. Although we cannot directly assess its influence on our results, the temperature-driven changes in diet performance are inconsistent with these responses and likely emerged despite them.

### Selective feeding and the potential adaptive value of dietary shifts

Tadpoles allowed to feed selectively assimilated a slightly higher proportion of animal-based food, suggesting a carnivory bias. However, consistent with our prediction, food assimilation changed with temperature, as the contribution of the animal-based food at 12 °C was higher compared to higher temperatures. This pattern followed a non-linear response, similar to trends reported in the literature^16,17^, and may reflect temperature-dependent changes in the relative nutritional value, digestibility, or assimilation efficiency of the two food items. Cold conditions promote a more efficient digestion of nitrogen-rich diets by increasing gut retention time while simultaneously suppressing microbial fermentation, thereby reducing the assimilation efficiency of plant-based diets^94^. These processes may explain the variation in diet quality with increasing temperature^16,17^, which favour dietary shifts towards herbivory and the increased assimilation of plant-based food detected in multiple ectotherms^25,27,30,32,33,95^. Nevertheless, species-specific morphology (i.e. relatively short guts^96)^ may hamper the efficient use of plant-based food and limit the capacity of *B. spinosus* tadpoles to perform large dietary shifts, cancelling the positive effects of plant-based diets on performance with increasing temperature, as seen in *D. galganoi*^27^. Yet, the magnitude of the dietary shifts detected here may be conservative relative to what may happen in nature, as the experimental setup did not account for the energetic trade-offs associated with prey search and pursuit, nor for the variable effects of increasing temperature on feeding rates according to predator-prey size ratio^11^. Furthermore, we used stable isotopes to estimate the assimilation of plant- and animal-based foods. This method is commonly employed in dietary studies^63^ but does not partition temperature effects on resource consumption and assimilation. Complementary measurements of resource consumption would allow more precise inferences on feeding behaviour and a better understanding of the potential ecological impacts.

The perceived changes in the assimilation of the plant- and animal-based foods point to selective feeding according to temperature, which may hold adaptive value. As predicted, contingent on temperature, tadpoles allowed diet choice generally outperformed tadpoles on the two diets with no choice, with their trait performance being subpar only for body condition at 12 °C. Notably, tadpoles on the choice diet maintained both body mass (16–20 °C) and body length (12–20 °C) across the experimental temperatures, unlike those on the animal- and plant-based diets. This greater trait performance of tadpoles able to feed selectively was expected, given that access to complementary foods improves nutritional balance and supports growth and earlier metamorphosis in anurans, and with improved performance in ectotherms in general^15,21,83,97^. In particular, the stabilizing effect of selective feeding may be of critical importance, as body length and mass are both traits linked to starvation tolerance and predator escape ability^35,36^.

Increasing temperature induced a stronger increase in the body C:N ratio of toadlets from the plant-based diet compared to toadlets from the two diets with animal-based food (A and C). This was not expected, given that changes in body stoichiometry are common in producers^98,99^, but less frequently reported in consumers^100,101^. Although the nutrient limitation experienced by herbivores consuming high C:N ratio diets can be reflected in their body stoichiometry^9^, omnivores and carnivores often maintain rather stable body C:N ratios^102^. Hence, the increase in body C:N ratio of the toadlets from the choice diet at warmer temperatures further supports a dietary shift towards herbivory and suggests that tadpoles experienced some physiological stress, being unable to maintain body stoichiometry. Additionally, this finding suggests the use of compensatory feeding as a secondary behavioural strategy to overcome nutrient limitation, which may cause an excessive accumulation of non-limiting nutrients^103^. Plasticity in consumer stoichiometry may hold adaptive value, allowing ectotherms to accommodate variation in nutrient availability, but can have wide repercussions in natural food webs by altering the quality of herbivore and omnivore consumers as a resource for predators or decomposers. Overall, this study suggests that while selective and compensatory feeding may have allowed tadpoles on the choice diet to buffer some trait-specific costs at the edges of the experimental thermal range, the modulation of temperature effects on their life-history became increasingly more difficult at higher temperatures.

## Conclusions

Our findings provide novel insights into the ability of ectotherms to use selective and compensatory feeding to buffer climate warming effects on trait performance and life-history architecture. Although ectotherm life-history architecture is likely constrained by taxon-specific evolutionary history and physiology, our results show that the life-history traits of *B. spinosus* were primarily governed by experimental temperature, which dictated the pace of development. Diet had a secondary and trait-specific role in shaping plastic responses, that was more evident for growth rate and body mass. Increasing temperature accelerated growth and developmental rate, reducing body mass and condition at metamorphosis, leading to smaller and leaner toadlets in agreement with the temperature-size rule. However, access to the two diet types enhanced tolerance to higher temperatures by partially offsetting its negative effects on trait performance. Cold conditions amplified the influence of diet on body mass by prolonging larval period and allowing for greater energy storage as fat or muscle tissue, maximized on the nitrogen-rich diet. This highlights that the benefits of rapid development at higher temperatures came at the cost of reduced body mass and condition. Although selective feeding and the consumption of balanced diets can mitigate some trait-specific costs under contrasting thermal conditions, the benefits of these behavioural strategies appear to be strongly constrained by life-history configuration. Responses to increasing experimental temperatures were context-dependent and the plasticity associated with the diet modulation of temperature effects was trait-specific. While some traits were plastic and highly responsive to diet (growth rate and body mass), others were largely driven by temperature (larval period). Hence, ectotherms may be unable to fully mitigate the effects of climate change via modulation of their diet according to temperature, as higher temperatures channel life-history, narrowing the breadth of diet-induced plasticity on trait performance. Understanding how temperature-diet interactions govern ectotherm nutrition and the performance landscapes for life-history traits is vital to predict warming impacts on species and ecosystems, and particularly urgent in species-rich, ectotherm-dominated, and highly threatened freshwater ecosystems.

## Supplementary Information

Below is the link to the electronic supplementary material.

Supplementary Material 1

## Data Availability

Data are available and accessible upon reasonable request (10.5281/zenodo.20426252).
