# Supplementary material for: Temperature constrains diet-induced plasticity in the life-history of an aquatic vertebrate
Source: Sci Rep. 2026 Jun 8;16:21840. doi: 10.1038/s41598-026-55894-y (PMC13365529; doi:10.1038/s41598-026-55894-y)
Supplement: Supplementary file 1 — Supplementary Material 1 [file 41598_2026_55894_MOESM1_ESM.docx]

**Temperature constrains diet-induced plasticity in the life-history of an aquatic vertebrate**

**Table S1.** Isotopic signature and elemental ratio of the two food sources. Values are presented as mean ± 95% confidence intervals, calculated using the t-distribution. δ¹³C and δ¹⁵N values are expressed in ‰.

| **Food source** | **δ^13^C** | **δ^15^N** | **C:N** |
| --- | --- | --- | --- |
| Animal | -26.42  (-26.55 – -26.30) | 11.01  (10.47 – 11.61) | 5.41  (5.33 – 5.49) |
| Plant | -27.78  (-27.84 – -27.62) | 4.20  (3.60 – 4.81) | 7.74  (7.48 – 8.00) |

**Table S2.** Test statistics of the linear mixed models (LMMs) used in the analysis of tadpole initial measurements (body weight, body length, and total length).

|  | **Temperature** | | |  | **Diet** | | |  | **Temperature × Diet** | | |
| --- | --- | --- | --- | --- | --- | --- | --- | --- | --- | --- | --- |
|  | df | *X*^2^ | *P* |  | df | *X*^2^ | *P* |  | df | *X*^2^ | *P* |
| Weight | 2 | 0.303 | 0.859 |  | 2 | 0.166 | 0.921 |  | 4 | 1.217 | 0.875 |
| Body length | 2 | 2.794 | 0.247 |  | 2 | 0.183 | 0.913 |  | 4 | 1.358 | 0.852 |
| Total length | 2 | 0.528 | 0.768 |  | 2 | 0.524 | 0.770 |  | 4 | 2.026 | 0.731 |

**Table S3.** Summary of the fitted mixed-effects models used to analyse survival, life-history traits, and whole-body C:N ratio. For survival, parameter estimates correspond to log hazard ratios from Cox mixed-effects models; estimates for other traits are given on the linear predictor scale of the corresponding mixed-effects models as means with 95% confidence intervals. Intercept = trait estimate at 12 °C on the animal diet. Symbols: W = initial weight; S = social variable; C = choice diet; P = plant diet. N Box = 18 boxes; N Cup = number of cups.

| ***Predictors*** | **Survival**  **rate** | **Growth**  **rate** | **Larval**  **period** | **Body**  **mass** | **Body**  **length** | **Body**  **condition** | **C:N**  **ratio** |
| --- | --- | --- | --- | --- | --- | --- | --- |
| Intercept | - | 0.06 -0.24 – 0.36 | 5.44 5.35 – 5.52 | 5.40  5.19 – 5.58 | 13.50 12.64 – 14.35 | 5.64  5.48 – 5.79 | 1.29 1.26 – 1.33 |
| W | -43.08 -65.66 – -20.50 | -6.11 -8.98 – -3.25 | -3.23 -4.10 – -2.36 | -0.83 -2.68 – 1.03 | -6.51 -14.84 – 1.83 | -0.19 -0.94 – 1.32 | -0.14 -0.49 – 0.21 |
| S | - | 0.00 0.00 – 0.01 | - | 0.00  0.00 – 0.00 | 0.01 0.00 – 0.01 | 0.00  0.00 – 0.00 | 0.00 0.00 – 0.00 |
| 16°C | 0.36 -0.77 – 1.49 | 0.92 0.75 – 1.09 | -1.09 -1.15 – -1.03 | -0.08 -0.19 – 0.04 | 1.06 0.54 – 1.57 | -0.35  -0.48 – -0.22 | 0.01 -0.01 – 0.02 |
| 20°C | -1.52 -3.30 – 0.25 | 1.06 0.89 – 1.23 | -1.78 -1.83 – -1.72 | -0.40 -0.51 – -0.29 | 0.00 -0.51 – 0.51 | -0.39 -0.52 – -0.27 | 0.00 -0.01 – 0.02 |
| C | -0.95 -1.78 – -0.13 | 0.08 -0.09 – 0.25 | -0.02 -0.07 – 0.03 | 0.08 -0.03 – 0.19 | 0.73 0.21 – 1.25 | -0.11 -0.27 – -0.04 | 0.00 -0.02 – 0.02 |
| P | 0.12 -0.59 – 0.83 | -0.28 -0.45 – -0.11 | -0.04 -0.09 – 0.01 | -0.20 -0.31 – -0.08 | 0.03 -0.48 – 0.54 | -0.21  -0.34 – -0.07 | 0.02 0.01 – 0.04 |
| 16°C × C | - | -0.15 -0.37 – 0.08 | -0.05 -0.11 – 0.01 | -0.16 -0.31 – -0.01 | -0.97 -1.66 – -0.28 | 0.10  -0.07 – 0.27 | 0.00 -0.03 – 0.02 |
| 20°C × C | - | 0.19 -0.04 – 0.42 | -0.05  -0.11 – 0.01 | 0.04 -0.11 – 0.19 | -0.34 -1.04 – 0.37 | 0.13  -0.03 – 0.29 | 0.02 -0.01 – 0.04 |
| 16°C × P | - | 0.13 -0.10 – 0.36 | 0.05 -0.01 – 0.12 | 0.11 -0.05 – 0.26 | -0.24 -0.94 – 0.47 | 0.17 0.01 – 0.32 | 0.00 -0.02 – 0.03 |
| 20°C × P | - | 0.30 0.07 – 0.52 | 0.05 -0.01 – 0.11 | 0.21 0.06 – 0.36 | 0.05 -0.63 – 0.73 | 0.20  0.06 – 0.34 | 0.06  0.04 – 0.09 |
| **Random**  **effects** |  |  |  |  |  |  |  |
| σ^2^ | - | 0.07 | 0.01 | 0.03 | 0.49 | NA | 0.00 |
| τ_00 Box_ | - | 0.00 | 0.00 | 0.00 | 0.00 | - | 0.00 |
| τ_00 Cup_ | - | 0.00 | 0.00 | 0.00 | 0.10 | 0.00 | 0.00 |
| ICC | - | - | 0.18 | - | 0.17 | - | 0.55 |
| N _Box_ | 18 | 18 | 18 | 18 | 18 | - | 18 |
| N _Cup_ | 135 | 124 | 127 | 124 | 124 | 124 | 123 |
| Observations | 270 | 210 | 228 | 210 | 207 | 207 | 130 |
| Marginal R^2^ /  Conditional R^2^ | - | 0.820 / NA | 0.988 / 0.990 | 0.489 / NA | 0.235 / 0.366 | 0.465 / 0.465* | 0.600 / 0.822 |
| * Values from an equivalent model with constant dispersion (same fixed and random effects, dispformula = ~ NULL). | | | | | | | |

**Table S4.** Comparison of the test statistics obtained with the Cox proportional hazards regression and GLMMs and with the 10,000 permutations. For each trait and model term (Temperature, Diet, and Temperature × Diet), we report the observed p-value obtained using the manual assignment of tadpole initial body mass, as well as the percentage of random within-pair permutations of tadpole initial mass assignments yielding p < 0.05, and the median permutation p-value with the central 95% interval (2.5–97.5% quantiles).

|  | **Temperature** | **Diet** | **Temperature × Diet** |
| --- | --- | --- | --- |
| Survival | P = 0.113; 0.0%  0.129 [0.121–0.136] | P = 0.032; 59.6%  0.049 [0.039–0.058] | - |
| Growth rate | P < 0.001; 100%  0.000 [0.000–0.000] | P < 0.001; 100%  0.000 [0.000–0.000] | P = 0.005; 100%  0.005 [0.003–0.008] |
| Larval period | P < 0.001; 100%  0.000 [0.000–0.000] | P < 0.001; 100%  0.000 [0.000–0.000] | P = 0.004; 100%  0.002 [0.002–0.004] |
| Body mass | P < 0.001; 100%  0.000 [0.000–0.000] | P < 0.001; 100%  0.000 [0.000–0.000] | P < 0.001; 100%  0.000 [0.000–0.000] |
| Body length | P < 0.001; 100%  0.000 [0.000–0.000] | P = 0.047; 99.2%  0.041 [0.039–0.048] | P = 0.057; 75.5%  0.047 [0.038–0.057] |
| Body condition | P < 0.001; 100%  0.000 [0.000–0.000] | P = 0.190; 0%  0.187 [0.160–0.242] | P = 0.104; 0%  0.109 [0.100–0.131] |
| C:N ratio | P < 0.001; 100%  0.000 [0.000–0.000] | P < 0.001; 100%  0.000 [0.000–0.000] | P < 0.001; 100%  0.000 [0.000–0.000] |


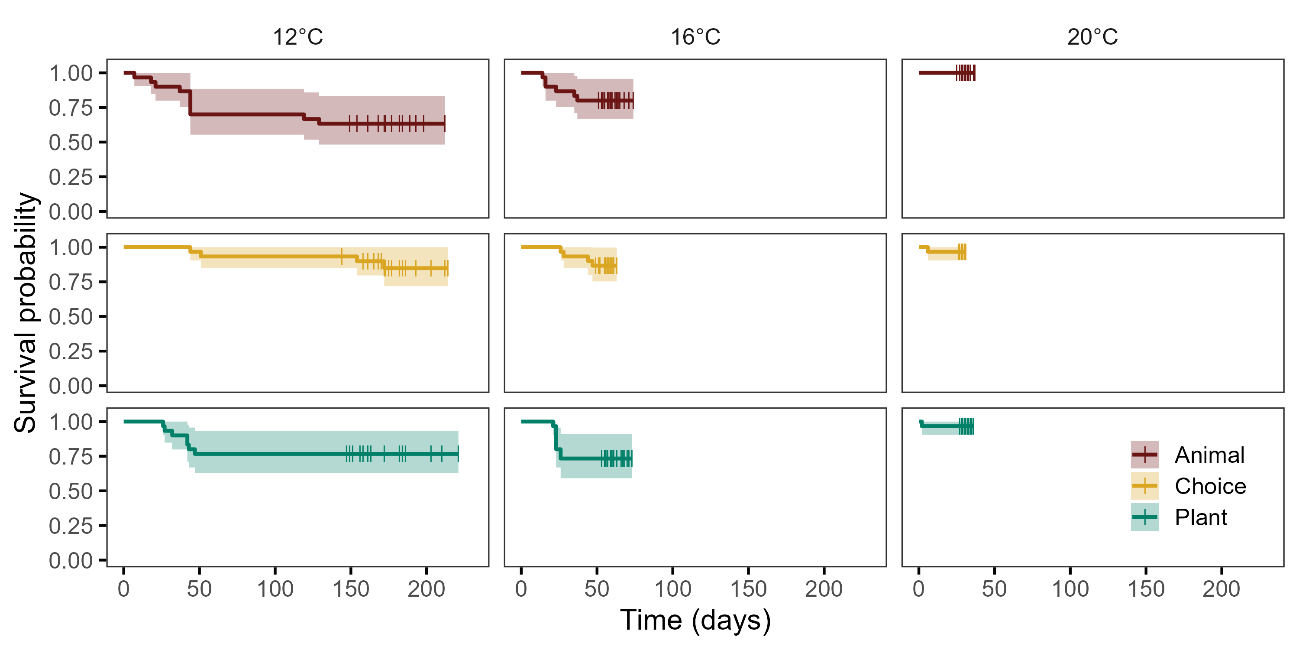


**Figure S1.** Cox proportional hazards regression analysis of the survival rate of *Bufo spinosus* tadpoles on animal (dark red), plant (teal), and mixed diets (yellow) at 12°C, 16°C, and 20°C. Lines with colour band represent mean fit ± 95% confidence intervals (CI), vertical dashes represent censoring time, i.e., time at which an individual was last seen alive (e.g., metamorphosis).


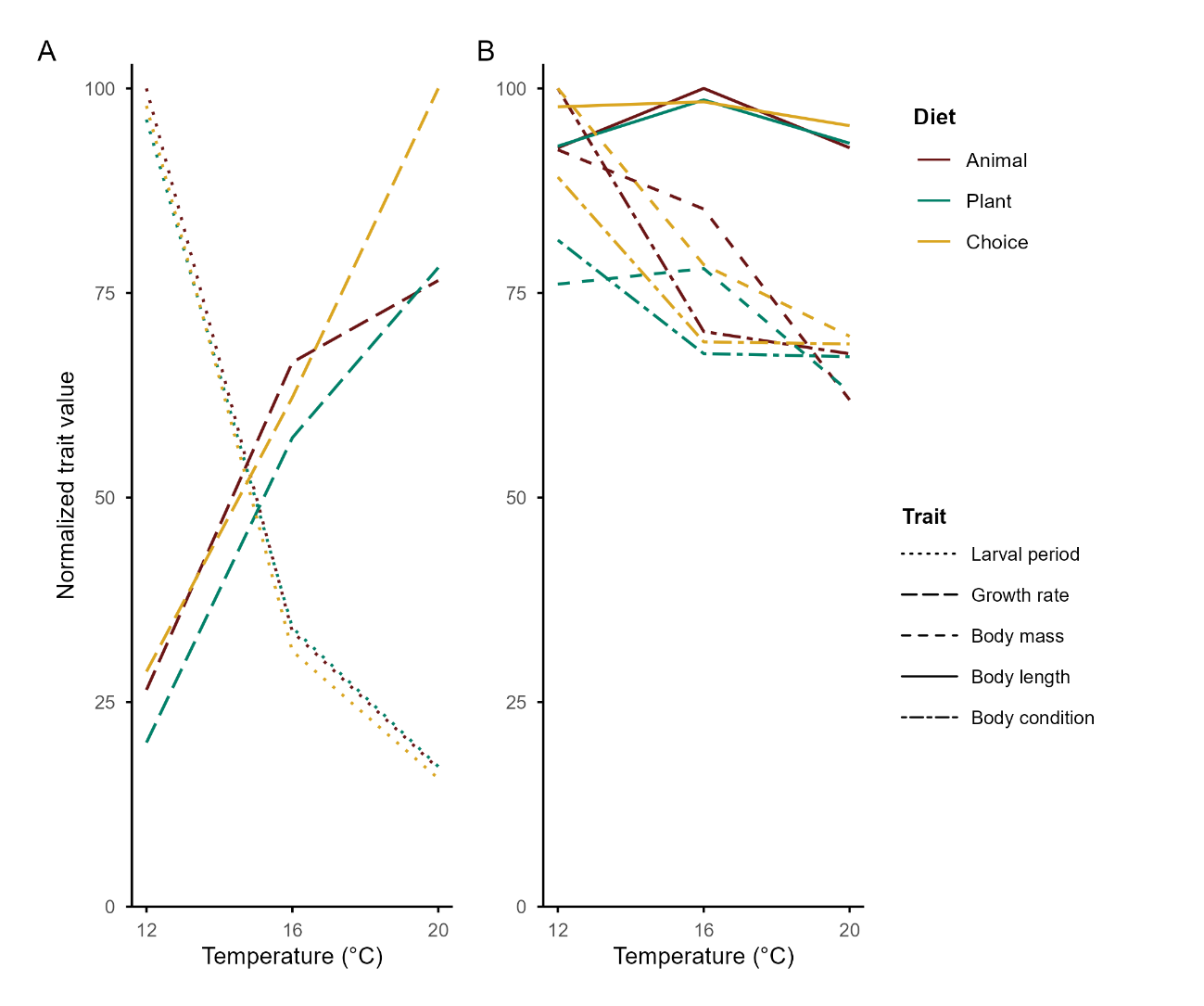


**Figure S2.** Lines represent model-estimated mean values on the animal (dark red), plant (teal), and choice diet (yellow) at 12 °C, 16 °C, and 20 °C, normalized to a 0–100 scale by dividing each trait by its highest treatment mean for each life-history trait of Bufo spinosus. Panel A) shows physiological traits: larval period (dotted line), and growth rate (long dash line); and panel B) shows toadlet morphological traits: body mass (dashed line), body length (solid line) and body condition (two-dash line).
